# Classifying hypopneas as obstructive or central can enhance transvenous phrenic nerve stimulation therapy patient selection and outcomes

**DOI:** 10.5664/jcsm.11904

**Published:** 2025-12-01

**Authors:** Kara Dupuy-McCauley, Alan R. Schwartz, Shahrokh Javaheri, Robin Germany, Scott McKane, Timothy I. Morgenthaler

**Affiliations:** ^1^ Center for Sleep Medicine, Division of Pulmonary and Critical Care Medicine, Mayo Clinic, Rochester, Minnesota; ^2^ University of Pennsylvania Perelman School of Medicine, Philadelphia, Pennsylvania; ^3^ Vanderbilt University School of Medicine, Nashville, Tennessee; ^4^ Universidad Peruana Cayetano Heredia, Lima, Peru; ^5^ SJMC Medical Group at the University of Maryland, Towson, Maryland; ^6^ Division of Pulmonary and Sleep, Bethesda North Hospital, Cincinnati, Ohio; ^7^ ZOLL Respicardia, Inc, Minnetonka, Minnesota

**Keywords:** central sleep apnea, central hypopnea, transvenous phrenic nerve stimulation

## Abstract

**Study Objectives:**

While not all sleep laboratories distinguish between obstructive and central hypopneas, recent research suggests that patients may receive an incorrect primary diagnosis without this effort. The remedē System Pivotal Trial studied transvenous phrenic nerve stimulation in patients with moderate-to-severe central sleep apnea. Entry criteria required apnea-hypopnea index (AHI) ≥ 20 events/h with central apnea index greater than obstructive apnea index and obstructive apneas < 20% of AHI but did not classify hypopneas. This analysis re-examined sleep studies from the trial to assess hypopnea classification impact on candidacy and treatment outcomes.

**Methods:**

Hypopneas were classified as central vs obstructive by a sleep core laboratory following a modified version of American Academy of Sleep Medicine recommended criteria. AHI composition was assessed pre/post treatment.

**Results:**

At baseline, 91% (138/151) of patients had ≥ 50% of events classified as central accounting for hypopnea classification. If all hypopneas were assumed obstructive, only 63% (95/151) would have had ≥ 50% central events. The likelihood of achieving ≥ 50% AHI reduction increased with the percentage of baseline events that were central: responder rates were 37.5% for patients with < 50% central events, incrementally increasing to 76.5% for those with ≥ 90% central events. At 6 months, residual AHI predominantly consisted of obstructive hypopneas. Central events decreased by 89% with treatment (baseline median 32 events/h). Obstructive apneas and hypopneas increased by 2 and 3 events/h, respectively.

**Conclusions:**

Distinguishing central from obstructive hypopneas is required to accurately determine the proportion of central and obstructive breathing events, and is crucial for appropriate therapy selection and managing patient expectations about treatment outcomes.

**Clinical Trial Registration:**

Registry: ClinicalTrials.gov; Name: Respicardia, Inc. Pivotal Trial of the remede System; URL: https://www.clinicaltrials.gov/study/NCT01816776, Identifier: NCT01816776.

**Citation:**

Dupuy-McCauley K, Schwartz AR, Javaheri S, Germany R, McKane S, Morgenthaler TI. Classifying hypopneas as obstructive or central can enhance transvenous phrenic nerve stimulation therapy patient selection and outcomes. *J Clin Sleep Med*. 2025;21(12):2113–2120.

**BRIEF SUMMARY**

**Current Knowledge/Study Rationale:** Hypopnea classification has been underused in sleep apnea diagnosis and may lead to patients receiving an incorrect diagnosis and/or appropriate treatment may be delayed. This analysis assessed how hypopnea classification of patients identified as having central sleep apnea may impact transvenous phrenic nerve stimulation candidate selection and treatment benefit.

**Study Impact:** Diagnosis of central sleep apnea was greatly affected by hypopnea classification. Excluding hypopneas or presuming all hypopneas are obstructive can greatly underestimate the presence of central sleep apnea. Importantly, the degree of effectiveness of transvenous phrenic nerve stimulation, which targets central sleep apnea, was informed by careful assessment of apnea and hypopnea event type burden.

## INTRODUCTION

Sleep apnea is a common condition in which there are repetitive breathing pauses that occur during sleep. Two major subtypes of sleep apnea exist: obstructive sleep apnea (OSA) and central sleep apnea (CSA). Since the mechanism by which episodes of apnea occur in CSA and OSA is different,^1^^–^^4^ treatment is not universal, and classification of each breathing event as obstructive or central is crucial in order to enhance diagnostic accuracy. Presence and severity of sleep apnea is currently defined by the apnea-hypopnea index (AHI), which is an assessment of 2 different kinds of disordered breathing events: apneas and hypopneas. Classifying apneas as obstructive or central is straightforward but this is not the case for hypopneas. The American Academy of Sleep Medicine (AASM) has proposed a visual method of classifying hypopneas as obstructive or central, but it has not been fully validated and existing data suggests limited accuracy and high interrater variability in how events are scored.^5^ Hypopneas are often not classified as obstructive or central due to lack of clarity on effective methods of classification, lack of awareness of impact of unscored hypopneas, and burden of increased time spent scoring. Furthermore, with the increased use of home sleep apnea testing, it is important to note that most home sleep apnea testing devices presume hypopneas to be obstructive by default. Without classification, patients may receive a primary diagnosis that does not reflect the disordered breathing phenotype, leading to suboptimal treatment decisions and potential barriers to accessing appropriate therapy.

Analytic scoring methods have evolved to extend our ability to distinguish central from obstructive events (hypopneas and apneas). The AASM method of classifying hypopneas states that a hypopnea should be classified as obstructive if one of the following is present: thoracoabdominal paradox, flattening of the inspiratory portion of the nasal pressure transducer curve, snoring during the event. If none of these is present, the hypopnea should be classified as central.^6^ Since the accuracy of this method may be limited, others have proposed additional factors to consider. Randerath et al created a visual algorithm that includes the AASM criteria with additional differentiating parameters to indicate an obstructive hypopnea: event occurs during stage rapid eye movement sleep, arousal from sleep occurs with the resumption of normal respiration, the termination of the event is sudden.^7^ However, when compared to the AASM criteria, this classification system was found to have similar accuracy and low interrater reliability.^5^ Building on these approaches, another method proposed by Javaheri, Rappoport, and Schwartz incorporates additional physiological characteristics to distinguish obstructive from central hypopneas.^8^

Classification of disordered breathing events has become even more important in recent years with the advent of implantable devices dedicated to treating only OSA *or* CSA.^9^^,^^10^ When considering an invasive treatment modality, it is imperative that the patient be accurately diagnosed so that they do not undergo an unnecessary surgery and device implantation and so that they can be effectively counseled as to the predicted efficacy of the implanted therapy.^11^^,^^12^ The remedē System is a commercially available implantable transvenous phrenic nerve stimulation (TPNS) device that treats moderate to severe CSA in adult patients. The device includes an implanted pulse generator placed in the chest (similar to a pacemaker) and a stimulation lead placed in a vein near the left or right phrenic nerve. The stimulation lead sends signals via the phrenic nerve to the diaphragm to initiate a breath. The therapy activates automatically at night when the programmed conditions for scheduled sleep time, position, and activity are met. Notably, the remedē device is designed to treat centrally mediated events by generating contraction of the diaphragm, however it does not improve upper airway patency and so is ineffective for OSA.

TPNS has shown promise in treating CSA based on apnea classification alone, but the impact of hypopnea classification on diagnosis and therapeutic outcomes is not clear. We hypothesized distinguishing central from obstructive hypopneas can enrich TPNS candidate selection and better predict response to therapy. To address this hypothesis, we reanalyzed data from the remede pivotal trial, which was a randomized trial finding TPNS therapy reduced the AHI by 25 events/h and nearly eliminated the central apnea index (CAI) (ClinicalTrials.gov identifier: NCT01816776).^13^^–^^15^ However, many patients still exhibited a mild to moderate level of residual sleep-disordered breathing events while on therapy. In the original analysis, hypopneas were not classified as central vs obstructive. For the present analysis, the original scoring was updated to differentiate the hypopneas as central, obstructive or mixed to enable examination of the composition of the baseline and residual AHI on treatment, and to predict the level of therapeutic success.

## METHODS

### Design

This retrospective analysis examined polysomnogram data from individuals in the remedē System Pivotal Trial. During the trial, individuals meeting entry criteria, including optimal medical management for any comorbidities, were implanted with the TPNS device and randomized to the TPNS group (therapy activated 1 month post implant) or Control group (therapy remained off during the 6 months randomized period). More detail on the trial design can be located in prior publications.^13^^,^^14^

In-laboratory attended polysomnography was performed at baseline to verify the eligibility criteria including AHI ≥ 20 events/h of sleep, ≥ 30 events/h central apneas, central apneas ≥ 50% of all apneas, and OAI < 20% of AHI. Hypopneas were not classified as obstructive or central during the original scoring by a sleep core laboratory (Registered Sleepers, Inc., Winter Haven, FL). For the purposes of this analysis, another sleep core laboratory (Sleep Center Services, St. George, UT) was provided the original scoring and asked to classify the hypopneas as central, obstructive, or mixed. At the 6-month visit, individuals completed a second polysomnogram that followed the same scoring process as described for the baseline studies. At both sleep core laboratories, a single scorer performed all scoring following a manual defining the rules for each event type.

The protocol was approved by local ethics or institutional review boards and all individuals provided written informed consent to participate in the study. The investigation conforms with the principles outlined in the Declaration of Helsinki.

### Polysomnogram scoring

Initial event scoring followed the 2007 AASM guidelines using the 4% rule. In the updated scoring, hypopneas were classified based on the AASM criteria and select parameters proposed by Javaheri/Rappoport/Schwartz (**Table 1**).^8^

**Table 1:** Characteristics of obstructive and central hypopneas used to classify hypopneas.

Characteristics Favoring Obstructive Hypopnea	Characteristics Favoring Central Hypopnea
There is snoring during the event (and not noise from transvenous phrenic nerve stimulation) There is inspiratory flattening of the nasal pressure flow signal compared to baseline breathing, and/or negative effort dependence (an early peak in inspiratory airflow followed by a roll-off in flow thereafter) And one of the following: ○ Thoracoabdominal paradox that occurs during the event but not during pre-event breathing ○ Increasing respiratory effort during the event without a concomitant increase in flow (a dissociation of respiratory effort and inspiratory airflow) ○ Abrupt termination of event with deep inspiration and concomitant restoration of airway patency ○ Progressive prolongation of the inspiratory duty cycle (time spent inhaling/total breathing cycle time) during the event	Synchronous changes in airflow and respiratory effort Symmetric (sinusoidal) fluctuations in all cardiorespiratory signals Parallel waxing and waning airflow, respiratory efforts, and breath sounds Crescendo snoring occurs after the event (rather than an abrupt resuscitative snort at event termination) Electroencephalogram arousal occurs at the peak of hyperpnea (rather than abruptly at event termination) Prolonged respiratory cycle length (> 25”) ≤ 2 flow limited breaths at the end of the hypopnea

A hypopnea event should be classified as obstructive if it meets the definition in the obstructive hypopnea column. A hypopnea event should be classified central if not obstructive and one of the characteristics in the central hypopnea column is true.

Hypopnea events were classified as central if the event did not meet the obstructive definition and if one of the central characteristics was present. Hypopnea events were classified as mixed if the event contained both central and obstructive features and if there were > 2 flow-limited breaths at the end of the event.

### Statistical methods

Descriptive statistics of the baseline AHI composition, including the hypopnea classifications, were calculated as median [first quartile, third quartile]. Various definitions of CSA were explored to assess the potential impact on sleep-disordered breathing diagnosis in this cohort, including the effect of assuming all hypopneas are obstructive in nature. Analysis of TPNS treatment results focused on the individuals randomized to TPNS therapy, with an aim to describe the residual AHI composition after 6 months of treatment and assess the impact of hypopnea classification on effectiveness (for example, do individuals with a higher overall percent of events being central have a better outcome). SAS 9.4 (SAS Manufacturer, SAS Institute Inc, Cary, North Carolina, USA) was used for all analyses.

## RESULTS

The remedē System Pivotal Trial included 151 randomized individuals (73 TPNS, 78 control). The average age was 65 years and individuals were primarily White (95%) and male (89%). Most individuals had at least 1 comorbidity, with cardiovascular conditions being the most prevalent: hypertension (75%), hyperlipidemia (74%), heart failure (64%), and coronary artery disease (56%).

**Figure 1:**
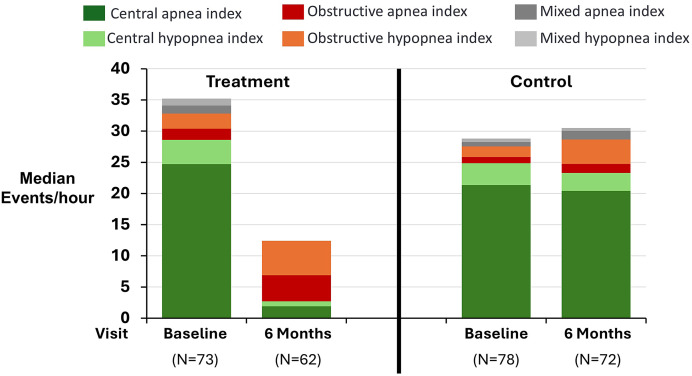
Summary of component medians by group at baseline and 6 months. The composition of the apnea-hypopnea index is displayed for each randomized group at each visit. In the treatment group, the central events were nearly eliminated with therapy. The composition of the apnea-hypopnea index did not change much in the control group at 6 months.

The median baseline sleep-disordered breathing metrics included AHI of 43.6 [31.8, 58.4] events/h, central AHI (CAHI) 30.1 [21.8, 41.5], obstructive AHI (OAHI) 4.4 [1.5, 11.6], and mixed AHI 2.7 [0.8, 6.5] (**Table 2**). The CAI (23.6 [14.5, 40.5]) was much higher than the central hypopnea index (3.7 [1, 9.1] events/h), whereas the obstructive apnea index and obstructive hypopnea index (1.5 [0.3, 3.4] vs 1.5 [0.4, 8.2] events/h) were similar in this CSA cohort. **Figure 1** displays the baseline composition of the AHI by treatment group. Interestingly, despite a median 79.5% [61.2, 91.6] of the events being central in nature, the percentage of hypopnea events that were central was only weakly correlated with the percentage of apneas that were central (Pearson correlation coefficient of 0.31). In general, individuals had a lower proportion of central hypopneas (48.9%) than central apneas (88.5%), and central hypopneas were more prevalent during non-rapid eye movement sleep, as expected.^16^

**Table 2:** Baseline polysomnogram characteristics.

Characteristic	All Enrolled (N = 151)
Apnea-hypopnea index (events/h)	43.6 [31.8, 58.4] (151)
Central apnea-hypopnea index (events/h)	30.1 [21.8, 41.5] (151)
Obstructive apnea-hypopnea index (events/h)	4.4 [1.5, 11.6] (151)
Mixed apnea-hypopnea index (events/h)	2.7 [0.8, 6.5] (151)
Central apnea index (events/h)	23.6 [14.5, 40.5] (151)
Obstructive apnea index (events/h)	1.5 [0.3, 3.4] (151)
Mixed apnea index (events/h)	0.9 [0.2, 3.4] (151)
Central hypopnea index (events/h)	3.7 [1, 9.1] (151)
Central hypopnea index during REM (events/h)	0 [0, 3.1] (135)
Central hypopnea index during NREM (events/h)	3.8 [1.1, 9.9] (151)
Obstructive hypopnea index (events/h)	1.5 [0.4, 8.2] (151)
Obstructive hypopnea index during REM (events/h)	4.2 [0, 17.1] (135)
Obstructive hypopnea index during NREM (events/h)	1.3 [0.3, 7.5] (151)
Mixed hypopnea index (events/h)	0.8 [0, 2] (151)
Percent of apneas and hypopneas central (%)	79.5 [61.2, 91.6] (151)
Percent of apneas central (%)	88.5 [73.4, 96.4] (151)
Percent of hypopneas central (%)	48.9 [19.6, 82.2] (146)

Characteristics from the baseline polysomnogram for the full study population demonstrate that while the proportion of apneas that were central was high (as required by the protocol), the proportion of hypopneas that were central was lower. Reported as median [first, third quartile] (n). NREM = non-rapid eye movement, REM = rapid eye movement.

The percentage of patients meeting various CSA definitions are displayed in **Table 3**. At baseline, 91% (138/151) of patients had ≥ 50% of events (apneas plus hypopneas) classified as central when accounting for hypopnea classification. If the common practice of assuming all hypopneas were obstructive had been employed, only 63% (95/151) of patients would have had ≥ 50% of their baseline events considered central. Additionally, 95% (144/151) of patients had CAHI ≥ 15 events/h, compared to 73% (110/151) with a CAI ≥ 15 events/h.

**Table 3:** Central sleep apnea definition analysis.

Characteristic	All Enrolled (N = 151) % (n)
CAI ≥ 5 events/h	100% (151)
CAHI ≥ 15 events/h	95% (144)
CAHI ≥ obstructive AHI	91% (138)
CAI ≥ 5 events/h and CAHI ≥ 50% of AHI	85% (128)
CAHI ≥ 15 events/h and CAHI ≥ 50% of AHI	84% (127)
CAI ≥ 15 events/h [note: this is the same result as CAI ≥ 15 events/h and CAI ≥ 50% of apnea index due to protocol requirement]	73% (110)
CAI ≥ 15 events/h and CAI ≥ 50% of AHI (assumes mixed apneas and all hypopneas are obstructive)	63% (95)

The definition used to determine the presence of central sleep apnea generates widely varying results, ranging from 100% when using CAI ≥ 5 events/h down to 63% using CAI ≥ 15 events/h and CAI ≥ 50% of AHI (assumes mixed apneas and all hypopneas are obstructive). AHI = apnea-hypopnea index, CAHI = central apnea-hypopnea index, CAI = central apnea index.

**Figure 2:**
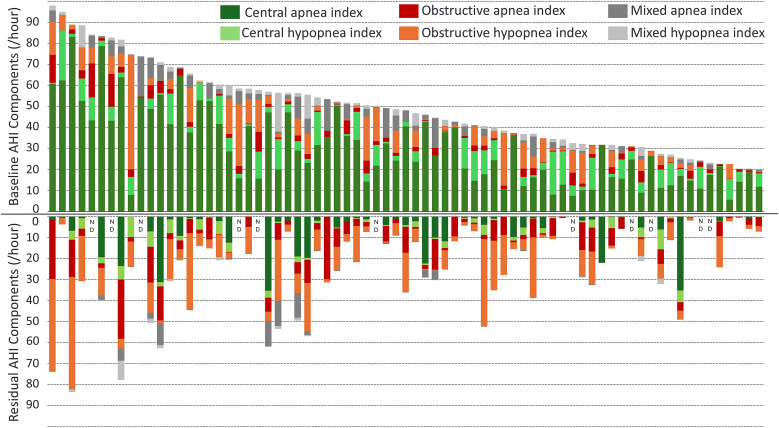
Breakdown of disordered breathing events by patient in the treatment group at baseline and at 6 months. The top half of the figure depicts the composition of the apnea-hypopnea index by event type at baseline, illustrating most of the events are central for nearly all patients, however after classification of hypopneas some patients had a large number of obstructive events. The bottom half of the figure depicts the residual event types after 6 months of therapy. The residual events are primarily obstructive apneas and hypopneas, which transvenous phrenic nerve stimulation is not designed to treat. AHI = apnea-hypopnea index.

After 6 months of treatment, the AHI, CAI and central hypopnea index were significantly reduced (**Figure 2**). The median residual AHI was reduced from 48.3 [32.1, 60.2] to 21.4 [11.3, 35.1] events/h, with the residual events primarily obstructive in nature (**Table 4**). The residual median CAI and central hypopnea index were 1.9 [0.3, 6.7] and 0.8 [0.2, 3.6] events/h, respectively, representing an 89% reduction in central events. Additionally, 81% (48/59) of individuals with CAHI ≥ 15 events/h shifted to CAHI < 15 events/h. In contrast, median residual obstructive apnea index and obstructive hypopnea index were 4.2 [1.7, 8.8] and 5.5 [1.9, 14.3] events/h, which were small increases from the baseline median. The mixed AHI reduced from 3.6 [1.1, 7] events/h at baseline to 0.2 [0, 0.9] events/h at 6 months.

**Table 4:** Treatment group polysomnogram characteristics by visit.

Metric	Baseline (N = 73)	Month 6 (N = 62)
Apnea-hypopnea index (events/h)	48.3 [32.1, 60.2] (73)	21.4 [11.3, 35.1] (62)
Central apnea-hypopnea index (events/h)	31.7 [21.2, 49.7] (73)	4.6 [1, 11] (62)
Obstructive apnea-hypopnea index (events/h)	5.2 [1.9, 11.8] (73)	11.1 [5.5, 25] (62)
Mixed apnea-hypopnea index (events/h)	3.6 [1.1, 7] (73)	0.2 [0, 0.9] (62)
Central apnea index (events/h)	24.7 [15.6, 41.6] (73)	1.9 [0.3, 6.7] (62)
Obstructive apnea index (events/h)	1.8 [0.5, 3.1] (73)	4.2 [1.7, 8.8] (62)
Mixed apnea index (events/h)	1.3 [0.4, 4.1] (73)	0 [0, 0.3] (62)
Central hypopnea index (events/h)	3.9 [0.9, 9.8] (73)	0.8 [0.2, 3.6] (62)
Central hypopnea index during REM (events/h)	0 [0, 4.1] (67)	0 [0, 0] (54)
Central hypopnea index during NREM (events/h)	4.0 [1, 10.3] (73)	0.9 [0.2, 4.2] (62)
Obstructive hypopnea index (events/h)	2.4 [0.3, 8.6] (73)	5.5 [1.9, 14.3] (62)
Obstructive hypopnea index during REM (events/h)	4.1 [0, 16.7] (67)	3.3 [0, 16.5] (54)
Obstructive hypopnea index during NREM (events/h)	1.5 [0.2, 8] (73)	4.8 [1.8, 15.6] (62)
Mixed hypopnea index (events/h)	1.1 [0.2, 2.1] (73)	0 [0, 0.2] (62)
Percent of apneas and hypopneas central (%)	78.1 [61.2, 90.4] (73)	20.4 [5.6, 43.6] (62)
Percent of apneas central (%)	87.8 [73.3, 94.5] (73)	29.4 [5.2, 60.3] (62)
Percent of hypopneas central (%)	42.2 [19.6, 75] (70)	18.2 [1.6, 35.6] (60)

The 6-month results demonstrate a significant reduction in the central apnea-hypopnea index and little change in the obstructive apnea-hypopnea index from baseline. Reported as median [first, third quartile] (n). NREM = non-rapid eye movement, REM = rapid eye movement.

**Figure 3:**
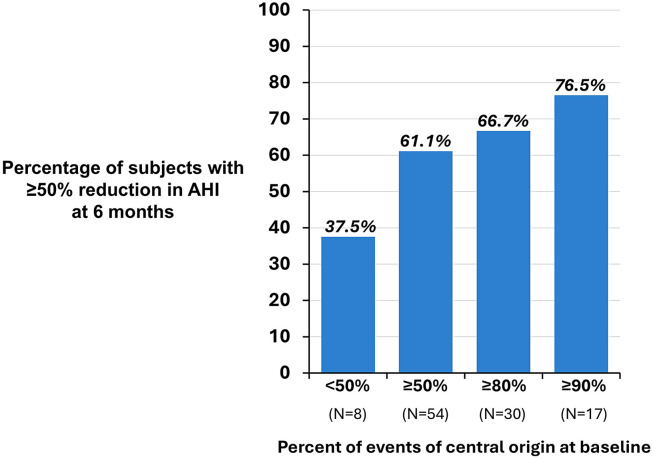
Impact of percentage of central events at baseline on effectiveness. The percentage of individuals achieving a 50% or higher reduction in the apnea-hypopnea index from baseline increased with the percentage of AHI that was central at baseline. AHI = apnea-hypopnea index.

**Figure 4:**
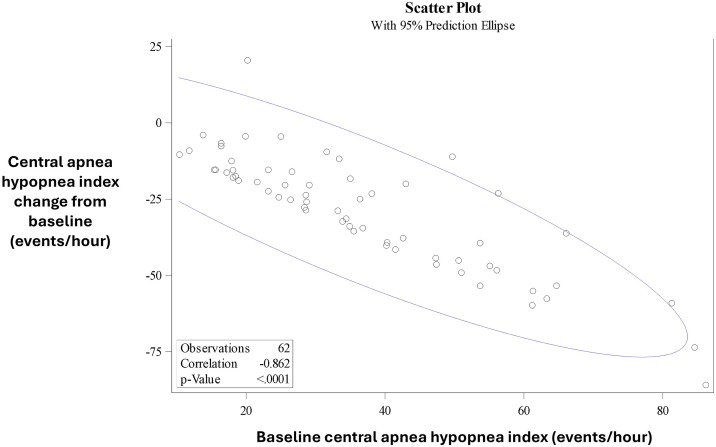
Correlation of baseline CAHI and change in CAHI. There was a high correlation (−0.862) between the baseline CAHI and the change in the CAHI after 6 months of therapy. Only 1 individual had an increase in the CAHI on therapy. CAHI = central apnea-hypopnea index.

The likelihood of achieving a ≥ 50% AHI reduction from baseline with TPNS therapy increased with the percentage of baseline events that were central. The responder rate was 37.5% (3/8) for patients with < 50% of their baseline events being central, incrementally increasing to 76.5% for those with ≥ 90% central events (**Figure 3**). Most central events were eliminated with therapy regardless of the initial frequency of disordered breathing events (**Figure 4**).

Exploratory analysis attempted to identify characteristics of patients who may be at risk of an increase in OAHI. Although no specific thresholds were determined, analysis suggested that higher baseline body mass index may suggest risk of increase in OAHI with TPNS therapy presumably due to connection between increased body weight and OSA. Individuals who experienced an OAHI increase of 5 or more events/h with TPNS therapy had a baseline median body mass index of 30.8 [27, 35.3] kg/m^2^ compared to 28.5 [26.8, 34] kg/m^2^ for individuals with < 5 events/h increase. The overlap of the body mass index distributions decreases the utility of this as a valuable predictor, although all patients with a body mass index > 40 kg/m^2^ failed to achieve an AHI reduction of at least 50%.

## DISCUSSION

Classification of hypopneas is essential to ensure that all patients who qualify for treatment of CSA are offered appropriate therapies. It also allows for accurate assessment of obstructive and central sleep-disordered breathing events, helping to avoid incorrect device placement in patients considering implantable therapies. Additionally, it improves the prediction of therapeutic outcomes, enabling thorough counseling on what patients can expect from treatment. Going forward, classifying hypopneas will allow us to study phenotypic response to TPNS, which will further enhance candidate selection. The high correlation of baseline CAHI and reduction in CAHI exemplifies the importance of classifying all of the events because TPNS is designed to treat central events and is not expected to effectively treat obstructive apnea or hypopnea events. Our analysis also suggests we cannot assume the proportion of central apneas directly reflects the proportion central hypopneas. We found approximately a 30 percentage point difference in the percentage of central apneas than the percentage of central hypopneas. Further, this analysis suggests the presence of residual hypopneas is due to most of the baseline hypopneas being obstructive and therefore not expected to respond to TPNS therapy. Indeed, when analyzing the central hypopneas using the updated scoring, central hypopnea index decreased from 3.9–0.8 events/h.

The key take-aways from this work is the significant impact hypopnea classification can have on defining eligibility for therapy and predicting patient outcomes. This classification is important in patients receiving noninvasive therapies so as not to delay treatment but is of utmost importance in patients who are being evaluated for invasive therapies, particularly in the setting of the new AASM clinical practice guideline addressing the treatment of CSA in adults. This guideline affirms the use of TPNS therapy in adults with primary CSA and CSA due to heart failure with a conditional recommendation for using TPNS over no TPNS. Patients with CSA considering TPNS.^17^ benefit from more precise candidate selection and predicting response to therapy including whether an additional treatment for OSA may be indicated. For example, there are current patients who have both a TPNS and an upper airway stimulation device,^11^ as well as patients using TPNS and continuous positive airway pressure devices or oral appliance therapy.^18^ Beyond therapeutic eligibility and treatment outcomes, hypopnea classification will likely allow for more accurate diagnosis and phenotyping of CSA, which has been one of the limitations in researching clinical outcomes. A panel recently convened to discuss CSA and underscored the need for future research to understand the clinical consequences of CSA and the impact that various therapies may have on these outcomes. Having an accurate diagnosis will be one of the first steps to facilitate accurate classification and allow us to work toward endotyping this diverse group of patients to gain more understand of the etiologies and pathophysiology of different subsets of patients with CSA.^19^

The initial remedē System pivotal trial results showed the hypopnea index remained relatively unchanged with TPNS therapy, despite further analysis suggesting patients with an AHI comprised of predominantly hypopneas experienced more than 50% reduction in AHI.^14^ Later analysis suggested patients with hypopnea index of ≥ 14 events/h had a higher response rate than those with < 14 events/h.^10^ Costanzo et al had suggested multiple reasons for the existence of residual events including limitations in ability to optimize voltage, be patient-initiated pauses in stimulation, and the gradual ramp leaving early events only partially addressed. But they also suggested the hypopneas present at baseline may have been obstructive, and therefore not impacted by this therapy targeting central events,^20^ and this is consistent with our findings. Along with confirming presence of obstructive hypopneas in these patients with predominantly central events, the observed small increase in OAHI after TPNS is employed suggests there may be some underlying narrowing of the upper airway. Phrenic nerve stimulation increases effective inspiratory effort, creating more negative intrathoracic pressure and higher inspiratory flow rates. This may increase upper airway collapsibility through a Starling resistor mechanism, potentially amplified by localized pressure drops associated with higher flow.

Patients with sleep-disordered breathing often have both central and obstructive events and only classifying apneas can overestimate the burden of OSA. A recent study illustrated that patients who primarily have OSA may have minimal amounts of CSA, however patients with CSA are more likely to have a significant OSA burden.^21^ It is not uncommon for diagnoses to be made either completely ignoring hypopneas or counting all hypopneas as obstructive events. Pepin et al examined how classifying hypopneas in a cohort of patients might influence the proportion of patients diagnosed with CSA and found that when hypopneas were classified, the percentage of patients with CSA increased 4-fold (from 5–20%).^21^

We identified several limitations with the current analysis. The method used to classify hypopneas as obstructive or central has not been validated for accuracy, interrater reliability, and sensitivity/specificity of classifying each type of disordered breathing event. A strength was the robust and varied methods to detect hypopneas, which we conjectured would add accuracy to hypopnea classification (although this has not been demonstrated in research). Robust number of individuals and inclusion of multiple etiologies of high loop gain CSA was also a strength. Given this was a retrospective post hoc analysis, the findings should be considered as hypothesis-generating.

## CONCLUSIONS

Distinguishing central from obstructive hypopneas is required to facilitate correct diagnosis in patients with sleep apnea, and is crucial for timely and appropriate treatment, optimal candidate selection, managing patient expectations regarding treatment outcomes, and to design future epidemiologic studies. This analysis suggests that accurate hypopnea classification may identify more eligible patients who could benefit from TPNS therapy, which effectively treats CSA syndrome, as well as aid in patient education about the possible need for treatment of residual OSA.

## DISCLOSURE STATEMENT

All authors have reviewed and approved this manuscript. The remedē System Pivotal Trial was sponsored by ZOLL Respicardia, Inc. K.D.: consultant. S.M. and R.G. are employees of ZOLL Respicardia, Inc. A.S. has been a consultant to ZOLL Respicardia, Inc. S.J. is a consultant for ZOLL Respicardia, Inc. T.M.: consultant.

## OPEN ACCESS

Copyright 2025 The Authors. This is an open access article, distributed under the Creative Commons Attribution 4.0 International License. Sharing and adaptation are permitted provided attribution to its original publication in the Journal of Clinical Sleep Medicine is made in accordance with the license.

## ABBREVIATIONS

AASM: American Academy of Sleep Medicine
AHI: apnea-hypopnea index
CAHI: central apnea-hypopnea index
CAI: central apnea index
CSA: central sleep apnea
OAHI: obstructive apnea-hypopnea index
OSA: obstructive sleep apnea
TPNS: transvenous phrenic nerve stimulation
